# Nutraceuticals, phytochemicals, and radical quenching ability of selected drought-tolerant advance lines of vegetable amaranth

**DOI:** 10.1186/s12870-020-02780-y

**Published:** 2020-12-14

**Authors:** Umakanta Sarker, Shinya Oba

**Affiliations:** 1 Department of Genetics and Plant Breeding, Faculty of Agriculture, Bangabandhu Sheikh Mujibur Rahman Agricultural University, Gazipur-1706, Bangladesh; 2 Laboratory of Field Science, Faculty of Applied Biological Sciences, Gifu University, Yanagido 1-1, Gifu, Japan

**Keywords:** Vegetable amaranth, Drought-tolerant, Nutraceuticals, Pigments, Phenolics, Flavonols, Flavanol, Flavones, Flavanones, Antioxidant activity, HPLC-UV, LC-MS-ESI, DPPH, ABTS^+^

## Abstract

**Background:**

Vegetable amaranth is a source of natural phytopigments and functional components of the commercial food industry for sustainable health benefits across the globe. It is guessed that recently identified amaranth (drought-tolerant) genotypes may contain ample phytopigments and phytochemicals suitable to extract juice as drinks. Hence, phytopigments and phytochemicals content of amaranth were assessed in detail for suitability as drinks to feed the phytochemicals deficient community across the globe.

**Results:**

The selected amaranth contained adequate carbohydrates, protein, moisture, and dietary fiber, phytopigments, minerals, phytochemicals including the ability to scavenge radicals. Nine flavonoids compounds were estimated in amaranth genotypes including six flavonols, one flavanol, one flavone, and one flavanone. It is the first effort in which we identified one flavonol such as myricetin, one flavanol, such as catechin, one flavone i. e., apigenin, and one flavanone, like naringenin in drought-tolerant vegetable amaranth. Across six flavonols, quercetin and rutin were the most noteworthy compounds followed by myricetin and isoquercetin. Across the accessions, AT7 and AT15 had abundant phytochemicals, and radical quenching ability including considerable proximate, nutraceuticals, and phytopigments in comparison to the accessions AT3 and AT11. AT15 demonstrated the maximum total flavonols including the highest rutin and hyperoside. AT7 showed high total flavonols including the highest quercetin, isoquercetin, myricetin, and kaempferol. The association of values revealed that studied phytopigments and phytochemicals of vegetable amaranth accessions demonstrated good radical quenching ability of 2,2-azino-bis (3-ethylbenzothiazoline-6-sulfonic acid) and 2,2- Diphenyl-1-picrylhydrazyl equivalent to Trolox.

**Conclusions:**

These advance lines AT7 and AT15 had abundant nutraceuticals, phytopigments, and phytochemicals including radical quenching ability. These lines might significantly contribute to the promotion of health benefits and feeding the community across the globe deficit in nutraceuticals and antioxidants. Identified flavonoid compounds open the new route for pharmacological study.

## Background

Vegetable amaranths are low-cost vegetables containing abundant nutraceuticals, minerals [1–7], phytopigments [8, 9], phytochemicals [10] including the important capacity to quench radicals and predominantly influenced the commercial food industry [11–13]. It has excessive phenotypic divergence in Bangladesh and Asia [14] and diversified culinary usage.

Recently, natural antioxidants, especially from vegetables attain the interest of researchers and consumers. Phytopigments containing betacyanins, betaxanthins, chlorophylls, carotenoids, and phytochemicals including vitamin C, β-carotene, flavonoids, and phenolics are available natural antioxidants in vegetable amaranth [11, 12]. These bioactive compounds protect numerous diseases including atherosclerosis, cardiovascular diseases, emphysema, cancer, retinopathy, cataracts, arthritis, and neurodegenerative diseases [15–17].

Leafy vegetables are the principal origin of natural antioxidants. Generally, vegetables (leafy) are so susceptible to abiotic stress like drought. The exploitation of drought-tolerant genetic resources at various stages of growth is foremost for semiarid regions [18]. Drought stress limits the leafy vegetables production in the drought-prone area though restriction of growth of leafy vegetables because of oxidative stress by inhibiting the cell elongation and expansion, reducing turgor pressure, altering the energy from growth to synthesis of secondary metabolites, reducing the water uptake, decreasing the tissue water contents, reducing the photo-assimilation and metabolites for cell division, generating the excessive free oxygen radicals [19]. The major sign of drought is osmotic stress that disrupts the ion distribution and homeostasis in the cell [20]. Hence, people of drought-prone areas are deprived of natural antioxidants through the production and consumption of leafy vegetables. However, vegetable amaranth is tolerant of drought [19, 21–23] and salinity [24–27]. Furthermore, previous studies of amaranth have displayed that drought stress remarkably augmented the ash, energy, protein, dietary fiber, Cu, Ca, Mn, K, S, Na, Mg, B, Mo content, TFC, TPC, vitamin C, total carotenoids, TAC (DPPH), beta-carotene, TAC (ABTS), flavonoids, and phenolic acids [21, 22]. A certain level of drought stress (eustress) remarkably augmented these nutraceuticals, phytopigments, and phytochemicals without compromising the loss of foliage yield [21, 22]. In literature, researchers reported that drought stress augmented the beta-carotene content in *Brassica* species [28] and herbs [29], vitamin C in tomato [30], flavonoids and polyphenols in buckwheat [31], antioxidant capacity, flavonoids, and polyphenols composition in *Achillea* species [32] antioxidant enzymes activity in walnut leaf [33], seedling [34], sugars and proline in radicle and plumule [35]; shoots and roots [36] of tolerant walnut.

Free radicals-induced injury predominantly depends on its equilibrium between formation and quenching through the antioxidant detoxification system [37]. Secondary metabolites synthesis like phytochemicals, phenolics, and flavonoids in stress induced-plants has excellent protection systems against free radicals to adjust the damage of oxidative stress [38]. These metabolites can quench free radicals in plants, as well as intake in the diet also have a significant role to cure many human oxidative damage and aging-related diseases [39].

In the previous studies, this research group screened vegetable amaranth germplasms based on agronomic performance, TAC, and yields compared with high yielding and antioxidant enrich existing popular cultivars and identified several high antioxidants enrich and high yield potential genotypes [1–5, 7, 40]. These identified genotypes were again screened against drought-stress to recognize the drought-tolerant accessions (Data not published). However, the nutraceuticals, phytopigments, and phytochemicals of antioxidants enrich drought-tolerant genotypes not assessed yet. It is guessed that the selected antioxidants enrich genotypes (tolerant to drought stress) may contain adequate nutraceuticals, phytopigments, and phytochemicals suitable to extract juice as drinks. Hence, the study was aimed to assess the chance of utilizing high yielding and antioxidant enrich amaranth as a source of natural nutraceuticals, phytopigments, and functional components for the industry of foods. Therefore, nutraceuticals, phytopigments, and phytochemicals content of amaranth were explored in detail for suitability as drinks to feed the phytochemicals deficient community across the globe.

## Methods

### Materials

Department of GPB of BSMRAU provided the seeds of four drought-tolerant advance genotypes. The detailed information of the genotypes is given in Table 1.

**Table 1:** The detailed information of the genotypes utilized in the study

Genotype	AT3	AT7	AT11	AT15
English name	Vegetable amaranth	Vegetable amaranth	Vegetable amaranth	Vegetable amaranth
Scientific name	*Amaranthus tricolor*	*Amaranthus tricolor*	*Amaranthus tricolor*	*Amaranthus tricolor*
Category	Leafy vegetables	Leafy vegetables	Leafy vegetables	Leafy vegetables
Edible parts	Baby stems and leaves	Baby stems and leaves	Baby stems and leaves	Baby stems and leaves
Usage	Salad or boiled	Salad or boiled	Salad or boiled	Salad or boiled
Edible stage	30 days old	30 days old	30 days old	30 days old
Characteristics	Red stems and leaves	Red stems and leaves	Red stems and leaves	Red stems and leaves
Source	Collection	Collection	Collection	Collection
Distinctive remark	Drought tolerant	Drought tolerant	Drought tolerant	Drought tolerant

### Experimental site, design, and layout

The evaluation was done in three replicates using a completely randomized block design (RCBD) at BSMRAU from 1st to 31st March 2015 (categorized as a subtropical zone, AEZ-28, 24°23′ north latitude, 90°08′ east longitude). The type of soil of the growing place is silty clay with monthly mean temperatures during the cropping period (maximum 34.00 °C, minimum 19.00 °C, and average 26.50 °C) and average relative humidity 54%. Each genotype was grown in one m^2^ plot on 1st March maintaining the distance of the rows of 20 cm and plants distance 5 cm.

### Intercultural practices

The compost (10 ton/ha) was amended during land preparation. TSP, Urea, MP, and Gypsum were utilized @ 100, 200, 150, and 30 kg/ha, respectively [40]. Intercultural operations, such as thinning, irrigation, and weeding were provided as required. The edible leaves of 10 selected plants (thirty days old) were randomly sampled.

### Estimation of proximate composition

Crude fat, ash, fiber, moisture, protein contents, and energy (gross) were measured by applying the AOAC method [41]. Crude protein was assessed by the Micro-Kjeldahl method. Finally, nitrogen was multiplied by 6.25 to measure crude protein (AOAC method 976.05). The protein, moisture, ash, and crude fat (%) was deducted from 100 for calculating carbohydrate (g 100 g^− 1^ FW) [21, 22].

### Mineral composition estimation

The leaves (fresh) were dried in an oven at 70 °C for 24 h. Calcium, potassium, magnesium, iron, manganese, copper, and zinc were determined from leaf powder following the digestion method of nitric-perchloric acid [21, 22]. The digestion was performed with carborundum beads adding 40 ml HClO_4_ (70%), 400 ml HNO_3_ (65%), 10 ml H_2_SO_4_ (96%) and 0.5 g dried leaf sample. After digestion, the method of ascorbic acid was followed to measure P through dilution of the solution appropriately in triplicate. Ascorbic acid and antimony were poured into the yellow-colored complex solution for converting it to a blue-colored phosphomolybdenum complex [42]. The optical density was estimated by atomic absorption spectrophotometry (AAS) (Hitachi, Tokyo, Japan) at wavelengths of 285.2 nm (magnesium), 248.3 nm (iron), 76 6.5 nm (potassium), 324.8 nm (copper), 422.7 nm (calcium), 213.9 nm (zinc), 279.5 nm (manganese).

### Determination of chlorophylls and carotenoids

Chlorophyll *ab*, chlorophyll *b*, carotenoids, and chlorophyll *a* were calculated by extracting the leaves in acetone (80%) [22, 43]. The optical density was taken using a spectrophotometer (Hitachi, Japan) at 646, 470, 663 nm for chlorophyll *b*, carotenoids, and chlorophyll *a*, respectively.

The formulae [44] were given below:

$$ \mathrm{Chlorophyll}\ a\left(\upmu \mathrm{g}/\mathrm{mL}\right)={\mathrm{C}}_a=12.21{\mathrm{A}}_{663}-2.81{\mathrm{A}}_{646} $$

Chlorophyll *b *(μg/mL) = C_*b*_ = 20.13 A_646_ − 5.03 A_663_

$$ \mathrm{Carotenoids}\left(\upmu \mathrm{g}/\mathrm{mL}\right)=\left(1000\ {\mathrm{A}}_{470}-3.27\ {\mathrm{C}}_a-104\ {\mathrm{C}}_b\right)/229 $$

Where: A_646_ = absorbance at a wavelength of 646 nm; A_663_ = absorbance at a wavelength of 663 nm; A_470_ = absorbance at a wavelength of 470 nm.

Finally, chlorophylls were calculated as micrograms per gram and carotenoids milligrams per 100 g of fresh weight, respectively.

### Betacyanins and betaxanthins content measurement

The leaves were extracted in methanol (80%, with 50 mM ascorbate) to estimate betacyanins and betaxanthins [22, 45]. The optical density was taken using a spectrophotometer (Hitachi, Japan) at 540 and 475 nm for betacyanins and betaxanthins, respectively. The data were calculated as nanograms betanin equivalent per gram of fresh weight for betacyanins and nanograms indicaxanthin equivalent per gram of fresh weight for betaxanthins [46].

### Estimation of beta-carotene

Ten ml acetone (80%) was mixed with 0.5 g leaves (fresh) and ground thoroughly. After centrifugation of the extract for 3–4 min at 10,000×g, the supernatant was removed and the volumetric flask was marked at the final volume of 20 ml [22, 47]. The optical density was measured with a spectrophotometer (Hitachi, Japan) at 480 and 510 nm. Finally, the results were quantified as mg beta-carotene per 100 g FW.

Beta-carotene = 7.6 (Abs. at 480) - 1.49 (Abs. at 510) × Final volume/(1000 × fresh weight of leaf) [48].

### Estimation of ascorbic acid

By pre-incubating and reducing the DHA of samples using Dithiothreitol, the ascorbic acid was determined. AsA reduced Fe^3+^ to Fe^2+^ ion. Fe^2+^ form complexes by binding with 2, 2-dipyridyl. The optical density of complexes was read by a spectrophotometer at 525 nm. The results were expressed as mg 100 g^− 1^ FW [22, 49].

### Samples extraction for TF, TP, and TAC

Leaves were air-dried in a shade. The extraction was performed in a capped test tube by mixing forty ml of 90% aqueous methanol with 1 g of fresh leaves (for TF) and dried leaf powder (for TF and TAC) of each genotype. After shaking the test tubes on a water bath shaker for 1 h, the mixture was centrifuged at 10,000×g for 15 min and filtered through a 0.45 μm filter. The TF, TAC, and TP were measured from this extract.

### Total polyphenols (TP) estimation

The determination of TP was performed following the previous method [22] using the reagent of Folin-Ciocalteau. The optical density was measured with a spectrophotometer (Hitachi, Japan) at 760 nm. The data were expressed as GAE μg g^− 1^ FW.

### Estimation of total flavonoids (TF)

The TF estimation was performed following the protocol of Sarker & Oba [22] using the AlCl3 method. The optical density was measured with a spectrophotometer (Hitachi, Japan) at 415 nm. The data were expressed as RE μg g^− 1^ DW.

### Radical quenching capacity assay

The determination of radical quenching capacity (RQA) was performed following the DPPH radical scavenging assay [22, 50] and the ABTS method [22, 51]. The optical density was measured using a spectrophotometer (Hitachi, Japan) at 517 and 734 nm for DPPH and ABTS, respectively. The RQA (ABTS and DPPH) was determined following the equation:

RQA (%) = (OD_b_ - ODs/OD_b_) × 100.

Where, RQA = radical quenching capacity, OD_b_ = optical density (blank) [as a replacement of extract, one hundred fifty and ten μl methyl alcohol for ABTS and DPPH, respectively], and ODs = The optical density of samples tested. The data were measured as μg TEAC g^− 1^ DW.

### Flavonols, flavanols, flavones, and flavanones analysis through HPLC

The leaf samples were extracted following the protocol described by Sarker and Oba [47, 49].

Flavonols, flavanols, flavones, and flavanones in the leaf sample were estimated using a Shimadzu SCL10Avp (Kyoto, Japan) HPLC equipped with a detector, binary pump, and degasser following the method of Sarker and oba [47, 49]. Flavonols, flavanols, flavones, and flavanones were separated using a column (STR ODS-II, 150 × 4.6 mm I.D., Kyoto, Japan). Acetic acid (6% v/v) in water and acetonitrile were pumped @ 1 ml/min for 70 min by the binary mobile phase as solvent A and solvent B, respectively. The injection volume and temperature of the column were maintained at 35 °C and 10 μl, respectively. Flavonols, flavanols, flavones, and flavanones were continuously monitored by setting the detector at 280, 370, and 360 nm. The compound’s identification was performed by comparing the retention time and UV–Vis spectra with its individual standards. The flavonols, flavones, flavanols, and flavanones were quantified using calibration curves of respective standards and confirmed through evaluating mass spectrometry. The identified compounds were determined as mg kg^− 1^ FW.

#### Statistical analysis

The sample data were averaged replication-wise to obtain the replication mean. Statistix 8 software was used to analyze the data for analysis of variance (ANOVA) [52, 53]. Duncan Multiple Range Test at a 1% level of probability was used to compare the means. The results were reported as the mean ± SD.

## Results

The ANOVA revealed that amaranth demonstrated huge variations across the traits studied.

### Composition of proximate

**Fig. 1:**
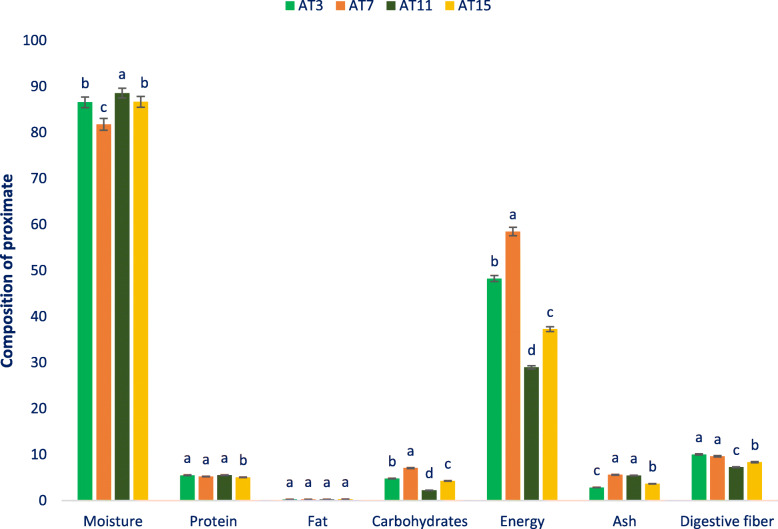
Composition of proximate (per 100 g^− 1^ FW) (energy kcal) in four selected drought-tolerant vegetable amaranth, different letters are differed significantly by Duncan Multiple Range Test (*P* < 0.01), (*n* = 6)

The composition of the proximate of selected amaranth is given in Fig. 1. The moisture of selected accessions varied from 81.79 to 88.56 g 100 g^− 1^ FW. The maximum content of moisture was noted in the advance line AT11 (88.56 g 100 g^− 1^ FW) followed by AT15 (86.68 g 100 g^− 1^ FW) and AT3 (86.57 g 100 g^− 1^ FW). On the contrary, the minimum moisture content was recorded in AT7 (81.79 g 100 g^− 1^ FW). Amaranth demonstrated considerable protein content that significantly varied regarding accessions albeit variability was not prominent (5.05 to 5.55 g 100 g^− 1^ FW). The maximum protein content was recorded in AT11 (5.55 g 100 g^− 1^ FW) which was statistically similar to AT3 and AT7. Conversely, the minimum protein content was noted in AT15 (5.05 g 100 g^− 1^ FW). Higher protein content was obtained from AT11, AT3, and AT7. In this investigation, amaranth demonstrated low-fat content.

The selected lines demonstrated significant variations in carbohydrates content regarding accessions (2.21 to 7.07 g 100 g^− 1^ FW). AT7 demonstrated the maximum carbohydrates content (7.07 g 100 g^− 1^ FW), while AT11 had the minimum carbohydrates content (2.21 g 100 g^− 1^ FW). The selected lines were predominantly varied with regard to energy (28.95 to 58.47 kcal 100 g^− 1^ FW). The accession AT7 demonstrated the maximum energy (58.47 kcal 100 g^− 1^ FW). Alternatively, the accession AT11 exerted the minimum energy (28.95 kcal 100 g^− 1^ FW). AT7 and AT11 demonstrated the maximum ash content (5.62 and 5.44 g 100 g^− 1^), respectively), while the minimum ash content was noted in AT3 (2.86 g 100 g^− 1^). Digestible fiber predominantly varied regarding accessions (7.26 to 10.03 g 100 g^− 1^ FW). The advance accession AT3 demonstrated the maximum digestible fiber content (10.03 g 100 g^− 1^ FW) followed by AT7. Conversely, AT11 had the minimum digestible fiber content (7.26 g 100 g^− 1^ FW).

### Mineral elements

**Fig. 2:**
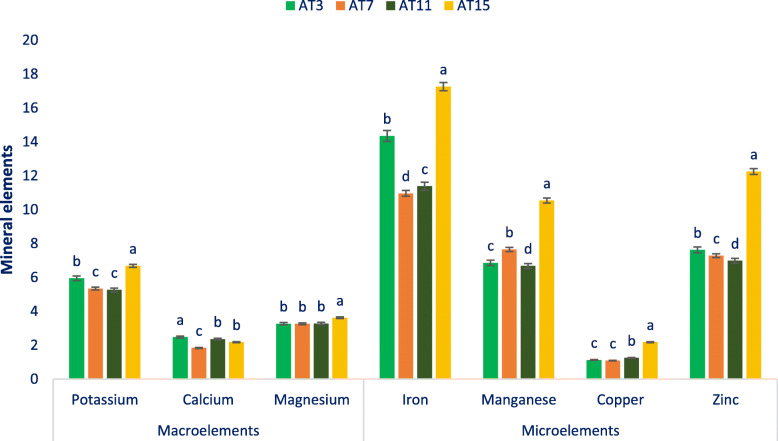
Mineral elements **(**Macroelements mg g^− 1^ FW, Microelements μg g^− 1^ FW) in four selected drought-tolerant vegetable amaranth, different letters are differed significantly by Duncan Multiple Range Test (*P* < 0.01), (*n* = 6)

Mineral elements of selected amaranth are put in Fig. 2. The lines demonstrated significant variability in potassium content regarding accessions. The maximum content of potassium was noted in AT15 (6.68 mg g^− 1^) followed by AT3, AT7, and AT11 including mean content of potassium of 5.81 mg g^− 1^. The maximum content of calcium was noticed in AT3 (2.48 mg g^− 1^) followed by AT11 and AT15. In contrast, the minimum content of calcium was noticed in AT7 (1.87 mg g^− 1^). In this investigation, the selected amaranth demonstrated significant variability in magnesium content regarding accessions (3.26 to 3.62 mg g^− 1^ FW)*.* AT15 demonstrated the maximum content of magnesium (3.62 mg g^− 1^ FW). Conversely, AT7 demonstrated the minimum content of magnesium (3.26 mg g^− 1^ FW) that had statistical similarity to AT3, AT11.

The iron content demonstrated significant variations regarding accessions (10.96 to 17.26 μg g^− 1^). The maximum iron content was obtained from AT15 (17.26 μg g^− 1^ FW), while the minimum iron content was noted in AT7 (10.96 μg g^− 1^ FW). It revealed from our study that significant variability in manganese content regarding accessions was noticed in amaranth (6.68 μg g^− 1^ FW and 10.54 μg g^− 1^ FW). Manganese content was the maximum in AT15 (10.54 μg g^− 1^ FW), while the minimum manganese content was recorded in AT11 and AT3 (6.68 and 6.86 μg g^− 1^ FW). The copper demonstrated no notable and significant variability in amaranth genotypes (1.09 to 2.18 μg g^− 1^ FW). AT15 demonstrated the maximum copper content (2.18 μg g^− 1^ FW), while AT7 exerted the minimum copper content (1.09 μg g^− 1^ FW). The zinc of amaranth differed significantly and markedly (6.98 μg g^− 1^ FW in AT3 to 12.25 μg g^− 1^ FW in AT15).

### Phytopigment contents

**Fig. 3:**
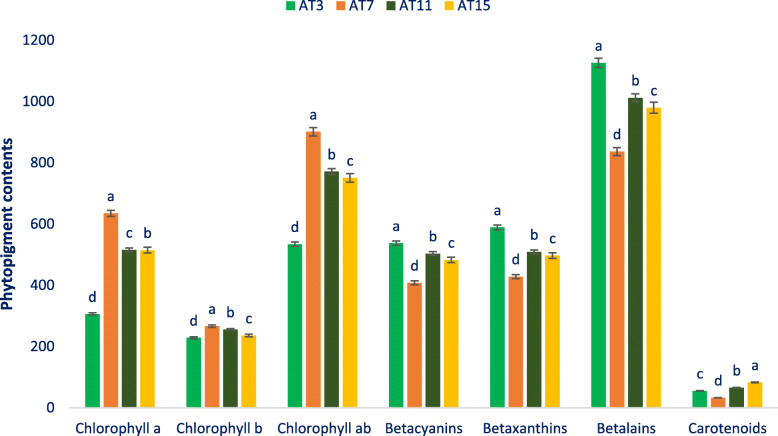
Phytopigments contents in four selected drought-tolerant vegetable amaranth, betacyanins (ng g^− 1^ FW), chlorophyll *a* (μg g^− 1^ FW), betaxanthins (ng g^− 1^ FW), chlorophyll *b* (μg g^− 1^ FW), betalains (ng g^− 1^ FW), chlorophyll *ab* (μg g^− 1^ FW), carotenoids (mg 100 g^− 1^ FW), different letters in the bar are differed significantly by Duncan Multiple Range Test ((*P* < 0.01), (*n* = 6)

Phytopigments of the selected amaranth are put in Fig. 3. The significant variability in chlorophyll *a* was noted regarding the selected amaranth (305.85 to 634.75 μg g^− 1^ FW). AT7 demonstrated the maximum chlorophyll *a* (634.75 μg g^− 1^ FW) followed by AT11 and AT15. Alternatively, the minimum chlorophyll *a* (305.85 μg g^− 1^ FW) was noted in AT3. Conversely, the selected amaranth demonstrated minimum differences in chlorophyll *b* (228.26 to 266.38 μg g^− 1^ FW). The maximum chlorophyll *b* was recorded in AT7 (266.38 μg g^− 1^ FW) followed by AT11. In contrast, AT6 demonstrated the minimum chlorophyll *b* (228.26 μg g^− 1^ FW). The significant variability in chlorophyll *ab* was recorded in amaranth genotypes (534.11 to 901.13 μg g^− 1^ FW). The advance line AT7 demonstrated the maximum chlorophyll *ab* (901.13 μg g^− 1^ FW) followed by AT11 and AT15, while AT3 demonstrated the minimum chlorophyll *ab* (534.11 μg g^− 1^ FW).

The selected amaranth demonstrated significant variability in betacyanin content regarding accessions (407.97 to 537.26 ng g^− 1^ FW). AT3 demonstrated the maximum content of betacyanins (537.26 ng g^− 1^ FW) followed by AT11 and AT15. In contrast, AT7 demonstrated the minimum content of betacyanins (407.97 ng g^− 1^ FW). The selected lines demonstrated significant variations in betaxanthins regarding accessions (428.45 to 588.75 ng g^− 1^ FW). AT3 demonstrated the maximum content of betaxanthins (588.75 ng g^− 1^ FW) followed by AT11 and AT15. Conversely, the minimum content of betaxanthins was noted in AT7 (428.45 ng g^− 1^ FW). The selected amaranth demonstrated significant variations in betalains content regarding accessions (836.42 to 1126.01 ng g^− 1^ FW). The AT3 demonstrated the maximum betalains (1126.01 ng g^− 1^ FW) followed by AT11 and AT15. While the AT7 demonstrated minimum betalains (836.42 ng g^− 1^ FW). Likewise, betalains, carotenoids showed significant variability in amaranth genotypes (32.75 to 82.85 mg 100 g^− 1^ FW). The maximum carotenoids were recorded in AT15 (82.85 mg 100 g^− 1^ FW). Whereas, AT7 demonstrated the minimum carotenoids (32.75 mg 100 g^− 1^ FW).

### Phytochemicals contents and radical quenching ability

**Fig. 4:**
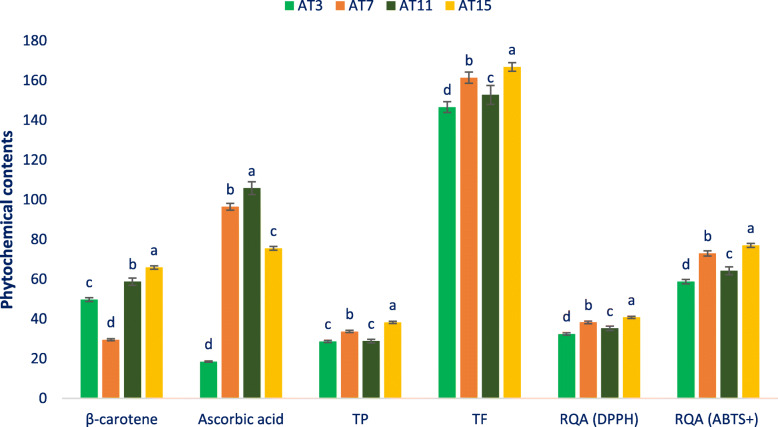
Phytochemical contents and radical quenching ability in four selected drought-tolerant vegetable amaranth, β-carotene (mg 100 g^− 1^ FW), ascorbic acid (mg 100 g^− 1^ FW), Gallic acid equivalent total polyphenols (TP, μg g^− 1^ FW), rutin equivalent total flavonoids (TF, μg g^− 1^ DW), Trolox equivalent radical quenching ability (DPPH) (μg g^− 1^ DW), Trolox equivalent radical quenching ability (ABTS^+^) (μg g^− 1^ DW); different letters in the bar are differed significantly by Duncan Multiple Range Test ((*P* < 0.01), (*n* = 6)

β-Carotene, ascorbic acid, total polyphenols (TP), total flavonoids (TF), and radical quenching capacity of amaranth are put in Fig. 4. The significant variability was recorded in the β-carotene of the selected amaranth (29.54 mg 100 g^− 1^ in AT7 to 65.87 mg 100 g^− 1^ in AT15). The high content of β-carotene.

was noticed in AT11. The lines demonstrated significant variability in ascorbic acid (18.54 to 105.78 mg 100 g^− 1^ FW). Ascorbic acid was the maximum in AT11 (105.78 mg 100 g^− 1^ FW) and the minimum in AT3 (18.54 mg 100 g^− 1^ FW). Marked and significant variability was recorded in gallic acid equivalent (GE) TP in the accessions (28.65 μg g^− 1^ to 38.23 μg g^− 1^ FW). AT15 demonstrated the maximum TP content (38.23 μg g^− 1^) followed by AT7. While AT3 demonstrated the minimum TP (28.65 μg g^− 1^) that had statistical similarity with AT11. The selected amaranth demonstrated rutin equivalent (RE) TF with significant variability regarding accessions (146.58 μg g^− 1^ to 166.81 μg g^− 1^ DW). AT15 demonstrated the maximum TF (166.81 μg g^− 1^) followed by AT7 and AT11, whereas AT3 had the minimum TF (146.58 μg g^− 1^). The selected amaranth demonstrated high DPPH and ABTS^+^ radical quenching ability. AT15 demonstrated the maximum Trolox equivalent (TE) DPPH and ABTS^+^ radical quenching ability (40.75, 76.98 μg g^− 1^) followed by AT7 (38.25, 72.96 μg g^− 1^). Alternatively, the minimum DPPH and ABTS^+^ radical quenching ability were recorded in AT3 (32.48, 58.75 μg g^− 1^) followed by AT11 (35.23, 64.23 μg g^− 1^).

### Flavonols, flavanols, flavones, and flavanones

The data of Table 2 represents the identified compounds, retention time, the molecular ion, main fragment ions in MS^2^, and λmax. The separated flavonols, flavones, flavanols, and flavanones compounds of four accessions of amaranth (AT3, AT7, AT11, and AT15) were compared with respective peaks and standard masses of these flavonols, flavones, flavanols, and flavanones. Nine flavonoids were detected in amaranth genotypes together with six flavonols, like kaempferol, rutin, hyperoside, myricetin, isoquercetin, and quercetin, one flavanol, like catechin, one flavone like apigenin, and one flavanone, such as naringenin. It is the first effort in which we identified one flavonols such as myricetin, one flavanol, such as catechin, one flavone i. e.,

**Table 2:** Retention time (Rt), wavelengths of maximum absorption in the visible region (λ_max_), mass spectral data and tentative identification of flavonols, flavanols, flavones, and flavanones in four selected drought-tolerant vegetable amaranth

Peak no	Rt (min)	λ_max_ (nm)	Molecular ion [M - H]^−^ (m/z)	MS^2^ (m/z)	Identity of tentative compounds
1	4.62	370	626.2142	626.2587	Myricetin-3-*O*-rutinoside
2	7.57	370	301.0487	301.0368	2-(3,4-dihydroxy phenyl)-3,5,7-trihydroxychromene-4-one
3	15.52	370	270.3524	270.3246	4′,5,7-Trihydroxyflavone, 5,7-Dihydroxy-2-(4-hydroxyphenyl)-4-benzopyrone
4	17.88	370	593.5264	593.3372	kaempferol-3-*O*-rutinoside
5	23.95	280	290.2341	290.2374	(2R-3S)-2-(3,4-dihydroxyphenyl)-3,4-dihydro-2-chromene-3,5,7-triol
6	26.76	280	271.0785	271.1742	Naringenin
7	54.38	360	463.3187	463.3421	Quercetin-3-*O*-glucoside
8	53.32	360	463.4584	463.5246	Quercetin-3-*O*-galactoside
9	53.35	360	609.3592	609.3623	Quercetin-3-*O*-rutinoside

apigenin, and one flavanone, like naringenin in amaranth genotypes. Figure 5 represents the identified flavonols compounds and Fig. 6 demonstrates the identified flavanols, flavones, and flavanones compounds of leaves of the selected amaranth. The four identified principal flavonoids groups showing the order: flavonols > flavanones > flavones > flavanols (Figs. 5 and 6). Across six flavonols, quercetin and rutin were quantified as the most prominent compounds followed by myricetin and isoquercetin in selected amaranth genotypes. Across the genotypes, AT15 demonstrated the maximum flavonols including the highest rutin and hyperoside. AT7 showed high total flavonols including the highest kaempferol, myricetin, isoquercetin, and quercetin. AT3 and AT11 contained low total flavonols including low kaempferol, rutin, hyperoside, myricetin, isoquercetin, and quercetin.

**Fig. 5:**
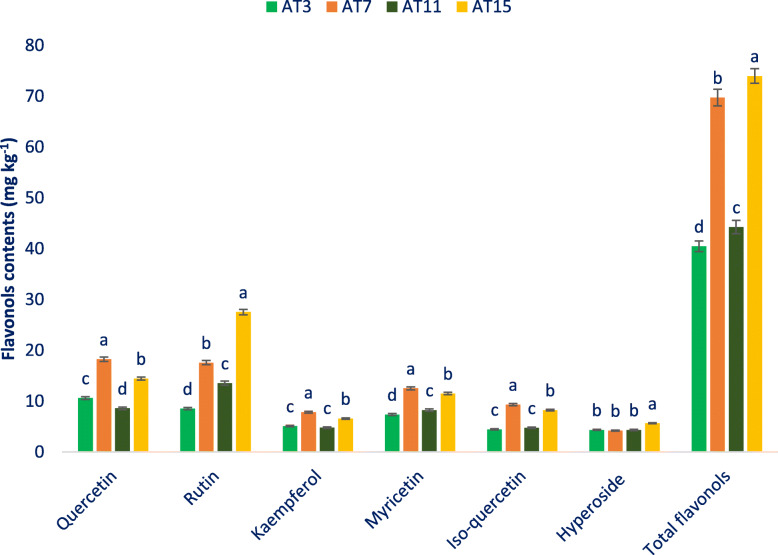
Flavonols contents (mg kg^− 1^ FW) in four selected drought-tolerant vegetable amaranth, different letters in the bar are differed significantly by Duncan Multiple Range Test ((*P* < 0.01), (*n* = 6)

**Fig. 6:**
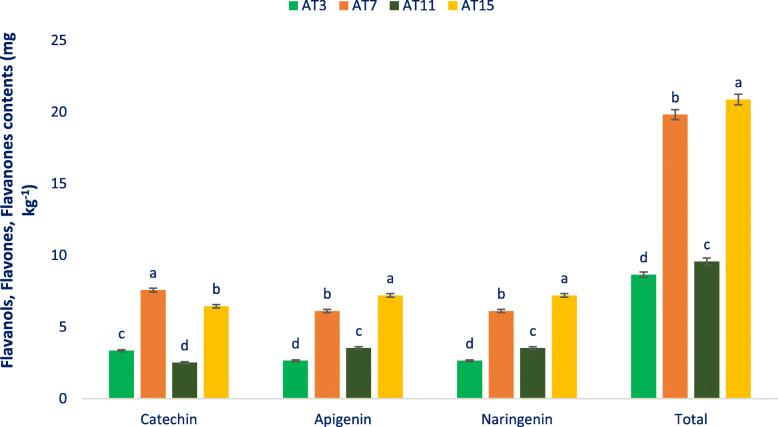
Flavanols, flavones, and flavanones content (mg kg^− 1^ FW) in four selected drought-tolerant vegetable amaranth, different letters in the bar are differed significantly by Duncan Multiple Range Test ((P < 0.01), (n = 6)

Kaempferol, rutin, hyperoside, myricetin, isoquercetin, and quercetin of selected amaranth genotypes varied from 4.78 to 7.82, 8.54 to 27.53, 4.22 to 5.66, 7.38 to 12.53, 4.45 to 9.34, and 8.59 to 18.25 mg kg^− 1^ FW, respectively (Fig. 5). AT7 demonstrated the maximum flavanols i. e., catechin followed by AT15. AT15 demonstrated the highest flavones i. e., apigenin, and flavanones, like naringenin followed by AT7. (Fig. 6). In contrast, AT11 had the minimum flavanols i. e., catechin followed by AT3. AT3 showed the minimum flavones i. e., apigenin, and flavanones, like naringenin followed by AT11 (Fig. 6).

### The correlation studies

The **c**orrelation of phytopigments, β-carotene, ascorbic acid, TF, TP, and radical quenching ability in ABTS^+^ and DPPH of the selected amaranth genotypes are put in Table 3. The correlation of phytopigments, β-carotene, ascorbic acid, TF, TP, and radical quenching ability in ABTS^+^ and DPPH of the amaranth genotypes demonstrated interesting results. All phytopigments positively and significantly correlated among TF, TP, and radical quenching ability in ABTS^+^ and DPPH. Similarly, ascorbic acid demonstrated a positive and significant interrelationship with TF, TP, and radical quenching ability in ABTS^+^ and DPPH, while it exhibited insignificant negative associations among all phytopigments.

**Table 3:** The coefficient of correlation for phytopigments, ascorbic acid, β-carotene, TP, TF, RQA (DPPH), and RQA (ABTS^+^) of four selected drought-tolerant vegetable amaranth

Traits	Chl *b* (μg g^− 1^ FW)	Chl *ab* (μg g^− 1^ FW)	BC (ng g^− 1^ FW)	BX (ng g^− 1^ FW)	BL (ng g^− 1^ FW)	β-Car (mg 100 g^− 1^ FW)	AA (mg 100 g^− 1^ FW)	TP (μg g^− 1^ FW)	TF (μg g^− 1^ DW)	RQA (DPPH) (μg g^− 1^ DW)	RQA (ABTS^+^) (μg g^− 1^ DW)
Chl*a* (μg g^− 1^ FW)	0.88**	0.93**	0.96**	0.95**	0.88**	−0.82**	−0.015	0.88**	0.87**	0.88**	0.95**
Chl*b* (μg g^−1^ FW)		0.95**	0.87**	0.95**	0.89**	−0.67	−0.018	0.81**	0.85**	0.92**	0.89**
Chl*ab* (μg g^−1^ FW)			0.82**	0.96**	0.87**	−0.85**	−0.014	0.86**	0.88**	0.86**	0.93**
BC (ng g^−1^ FW)				0.97**	0.95**	−0.86**	− 0.116	0.85**	0.87**	0.89**	0.88**
BX (ng g^−1^ FW)					0.97**	−0.85**	−0.123	0.83**	0.81**	0.85**	0.92**
BL (ng g^−1^ FW)						−0.89**	− 0.129	0.89**	0.86**	0.87**	0.89**
β-Car (mg 100 g^−1^ FW)							0.76*	0.89**	0.89**	0.92**	0.96**
AA (mg 100 g^−1^ FW)								0.86**	0.85**	0.87**	0.88**
TP (μg g^−1^ DW)									0.85**	0.88**	0.99**
TF (μg g^−1^ DW)										0.85**	0.89**
RQA (DPPH) (μg g^−1^ DW)											0.97**

*Chl a* Chlorophyll *a; Chl b* Chlorophyll *b; Chl ab* Chlorophyll *ab; BC* Betacyanins; *BX* Betaxanthins; *BL* Betalains; *β-Car* Beta-carotene; *AA* Ascorbic acid; *RQA (DPPH)* Radical quenching ability (TE); *RQA (ABTS*^*+*^*)* Radical quenching ability (TE); *TP* Total polyphenols (GAE); *TF* Total flavonoids (RE); ** Significant at 1% level

A significant positive association was exhibited among β-carotene, ascorbic acid, TF, TP, and radical quenching ability in ABTS^+^ and DPPH. Phytopigments and Phytochemicals including β-carotene, ascorbic acid, TP, and TF showed significant associations with radical quenching ability in ABTS^+^ and DPPH.

## Discussion

Nowadays, consumers and food researchers are fascinated by nutraceuticals and phytochemicals including vitamins, phytopigments, flavonoids, and polyphenols of plant origins, their antioxidant potentials, availability in diets, and activity of preventing fatal diseases viz., neuro-degenerative, cancer, and cardiovascular diseases [54]. Phytopigments, vitamins including β-carotene and vitamin C, flavonoids, and polyphenol compounds from natural origins, such as vegetables and fruits serve as antioxidants and protect several diseases [55]. Antioxidants compounds reduce oxidative damage to the body through inhibition of the oxidizing chain reactions caused by free radicals [56].

The ANOVA has shown that amaranth demonstrated huge variations across the traits studied. Huge variations across the traits studied were also noticed in *A. hypochondriacus* [42], rice [57–71], maize [72–74], and coconut [75, 76]. Across four drought-tolerant vegetable amaranth, only AT7 showed low moisture content. As more leaf mass (dry) was obtained from lower moisture contents, accessions AT7 (18% dry matter) had considerable biomass (dry). The maturity is directly interrelated to the leaf’s moisture content. Literature has shown the corroborative results in sweet potato [77] and *A. tricolor* [21]. Leaves of selected amaranth demonstrated adequate protein content that significantly varied regarding accessions albeit variability was not prominent. Poor people and vegetarians of low-income countries mainly depend on vegetable amaranth for their protein source. The protein of amaranth was much pronounced as compared to *A tricolor* (1.26%) of the previous study [2]. The lines exhibited low fat and might be consumed as a cholesterol-free food. The selected amaranth demonstrated no significant variability in fat content. Our results had conformity with the study of *A. tricolor* [21] and sweet potato [77]. The selected amaranth demonstrated significant variability in carbohydrates content regarding accessions. Digestible fiber significantly and predominantly varied regarding accessions. Digestible fiber remarkably contributed to the cure of constipation, increment of digestibility, and palatability [2]. Leaves of amaranth have abundant protein, moisture, carbohydrates, and digestible fiber. This study had conformity with our earlier study [21]. The moisture and protein contents observed in amaranth were pronounced than moisture and protein of red amaranth [78], green amaranth [79], weedy amaranth [80], stem amaranth [81], and *A. blitum* [82]. The carbohydrates content of AT7 were pronounced than the carbohydrates content of red amaranth [78], green amaranth [79], *A. spinosus* weedy amaranth [80], and *A. blitum* [82], while this line corroborated with carbohydrates content of stem amaranth [81]. The digestible fiber of the advance lines AT3 and AT7 were pronounced than the digestible fiber of red amaranth [78], green amaranth [79], stem amaranth [81], and *A. blitum* [82], while these lines corroborated with digestible fiber contents of weedy amaranth [80].

It revealed that we noted ample potassium (6.68 mg g^− 1^) and magnesium (3.62 mg g^− 1^), and Ca (2.48 mg g^− 1^) in the amaranth genotypes (based on fresh weight). Ample magnesium, calcium, and potassium were noted in several species of amaranth [83]. Additionally, they noticed that amaranth’s magnesium, calcium, and potassium were much prominent than black spider flower, nightshade, kale, and spinach. The magnesium, calcium, and potassium recorded in the selected amaranth were much pronounced than the magnesium, calcium, and potassium of amaranths [83] and fresh walnut [84]. The potassium of the lines was pronounced than the potassium of green amaranth [79], while potassium content obtained from these advance lines was lower than the potassium of weedy amaranth [80]. The calcium content observed in the genotypes were corroborative to green amaranth [79] and weedy amaranth [80]. The magnesium recorded in the advance lines was pronounced than green amaranth [79] and weedy amaranth [80]. The iron content demonstrated prominent variations regarding accessions. It has shown that significant variability was recorded in the manganese of the selected amaranth. The copper demonstrated no notable variability in the amaranth genotypes. The zinc of the selected amaranth differed significantly and markedly. The zinc and iron of the selected amaranth were more pronounced than cassava (leaf) [85] and beach pea [86]. It revealed from this investigation that ample iron (17.26 μg g^− 1^), manganese (10.54 μg g^− 1^), zinc (12.25 μg g^− 1^), and notable copper (2.18 μg g^− 1^) (based on fresh weight) were recorded in amaranth genotypes. Similarly, adequate iron, manganese, copper, and zinc were noticed in different amaranths [83]. They also noticed that manganese, iron, zinc, and copper in amaranth genotypes were pronounced than the flower of spider, kale, nightshade (black), and spinach. Our obtained manganese, zinc, iron, and copper content were much pronounced than the manganese, zinc, iron, and copper content of different amaranths [83]. The manganese and iron contents of all advance lines were much pronounced than green amaranth [79], while manganese and iron contents of AT15 were much pronounced than weedy amaranth [80]. Copper content observed in the lines was much pronounced than green amaranth [79], and *A. spinosus* weedy amaranth [80]. Zinc content observed in the advance line AT15 was much pronounced than green amaranth [79] and weedy amaranth [80].

The significant variability in chlorophyll *a* was noted regarding the selected amaranth (305.85 to 634.75 μg g^− 1^ FW). Conversely, the selected amaranth demonstrated minimum differences in chlorophyll *b*. Significant and outstanding variability in chlorophyll *ab* were recorded in the amaranth genotypes. We observed adequate chlorophyll *a* (634.75 μg g^− 1^ FW), chlorophyll *ab* (901.13 μg g^− 1^ FW), and chlorophyll *b* (266.38 μg g^− 1^ FW) in the amaranth genotypes which was pronounced than the chlorophylls of green and red amaranth [87]*.* Chlorophyll *a*, chlorophyll *ab*, and chlorophyll *b* in this study were much pronounced than chlorophyll *a*, chlorophyll *ab*, and chlorophyll *b* of red amaranth [78], green amaranth [79], weedy amaranth [80], stem amaranth [81], and *A. blitum* [82]. The lines demonstrated betacyanins, betalains, and betaxanthins with significant variability regarding accessions. Likewise, betalains, carotenoids showed significant variability in the amaranth genotypes. Our study demonstrated prominent chlorophyll *a* (634.75 μg g^− 1^ FW), chlorophyll *ab* (901.13 μg g^− 1^ FW), betacyanins (537.26 ng g^− 1^ FW), chlorophyll *b* (266.38 μg g^− 1^ FW), betaxanthins (588.75 ng g^− 1^ FW), betalains (1126.01 ng g^− 1^ FW) and carotenoids (82.85 mg 100 g^− 1^ FW) in amaranth genotypes which were corroborated with chlorophyll *a*, betacyanins, chlorophyll *b*, betalains, chlorophyll *ab*, betaxanthins, and carotenoids of green and red amaranth [87]*.* Betacyanins, betalains, and betaxanthins in the amaranth were much pronounced than betacyanins, betalains, and betaxanthins of red amaranth [78], green amaranth [79] stem amaranth [81], and *A. blitum* [82]. Carotenoids were much pronounced than carotenoids of green amaranth [79] and corroborated with weedy amaranth [80] while carotenoids of studied amaranth were lower than red amaranth [78], stem amaranth [81], and *A. blitum* [82].

The significant variability was recorded in the β-carotene of the selected amaranth. The amaranth demonstrated significant variability in ascorbic acid. Marked and significant variability was obtained in gallic acid equivalent (GE) TP in amaranth genotypes. The lines demonstrated rutin equivalent (RE) TF with significant variability regarding accessions. The selected amaranth demonstrated significant variations in DPPH and ABTS^+^ radical quenching ability. The similarity in the trend of radical quenching ability in DPPH and ABTS^+^ methods validated the quantification of two different methods of antioxidant capacities. The lines exhibited outstanding β-carotene and ascorbic acid (65.87 and 105.78 mg 100 g^− 1^ FW) which was pronounced than red amaranth [2]*.* TP (38.23 μg g^− 1^ FW), TF (166.81 μg g^− 1^ DW), radical quenching ability in DPPH (40.75 μg g^− 1^ DW), and radical quenching ability in ABTS^+^ (76.98 μg g^− 1^ DW) obtained in amaranth was pronounced than the contents of these compounds in green and red amaranth [51]. The β-carotene recorded in the advance lines was corroborative to weedy amaranth [80]. The ascorbic acid recorded in the line AT11 was pronounced as green amaranth [79], weedy amaranth [80], stem amaranth [81], and *A. blitum* [82], and corroborative to red amaranth [78]. The TP of amaranth was pronounced than green amaranth [79] and weedy amaranth [80]. The TF, radical quenching ability in DPPH and ABTS^+^ recorded in the advance lines was pronounced than red amaranth [78], green amaranth [79], weedy amaranth [80], stem amaranth [81], and *A. blitum* [82]. The lines AT15 and AT7 had high phenolics, flavonoids, and antioxidants including considerable nutrients, phytopigments, and vitamins in comparison to AT3 and AT11. These two accessions might be exploited as HYB cultivars containing ample antioxidant profiles and suitable to extract juice as drinks. It revealed from the investigation that the selected amaranth contained ample source of nutritional values, antioxidant phytochemicals, and antioxidant activity that put forward a large possibility to feed the community deficit in minerals, antioxidants, and vitamins.

Nine flavonoids were detected in the genotypes together with six flavonols, like kaempferol, rutin, hyperoside, myricetin, isoquercetin, and quercetin, one flavanol i. e., catechin, one flavone i. e., apigenin, and one flavanone, such as naringenin. For the first time, we identified one flavonol such as myricetin, one flavanol i. e., catechin, one flavone i. e., apigenin, and one flavanone, like naringenin in the amaranth genotypes. In green and red amaranth, three flavonols such as isoquercetin, hyperoside, and rutin were previously reported [51, 87]. Three flavonols, like kaempferol, rutin, and quercetin were previously recorded in the flowers, leaf, sprouts, seed, and stalks of several amaranth species (*A. cruentus*, *A. caudatus*, and *A. hypochondriacus*) [88]. The *A. cruentus* seeds and sprouts contained three flavonoids namely, rutin, isovitexin, and vitexin [89]. The four identified principal flavonoids groups of amaranth showing the order: flavonols > flavanones > flavones > flavanols. Across six flavonols, quercetin and rutin were quantified as the most prominent compounds followed by myricetin and isoquercetin in selected amaranth genotypes. Across the genotypes, AT15 exhibited the maximum flavonols including the highest rutin and hyperoside. AT7 showed high total flavonols including the highest kaempferol, quercetin, isoquercetin, and myricetin. AT3 and AT11 contained low total flavonols including low rutin, quercetin, isoquercetin, kaempferol, myricetin, and hyperoside. Hyperoside and quercetin of selected genotypes were pronounced than the hyperoside and quercetin content of red amaranth [51]. The edaphic and climatic situations, differences in cultivars, management practices, and geographic differential may be contributed a key role in achieving greater hyperoside and quercetin in the lines in comparison to red amaranth [51]. Pronounced variability was observed for kaempferol, rutin, hyperoside, myricetin, isoquercetin, and quercetin of the selected amaranth genotypes. AT7 demonstrated the maximum flavanols i. e., catechin followed by AT15. AT15 demonstrated the maximum flavones i. e., apigenin, and flavanones, like naringenin followed by AT7.

The correlation of phytopigments, β-carotene, ascorbic acid, TF, TP, and radical quenching ability in ABTS^+^ and DPPH of the selected genotypes demonstrated interesting results. All phytopigments positively and significantly correlated among TF, TP, and radical quenching ability in ABTS^+^ and DPPH. It indicated that the increment in TF, TP, and radical quenching ability in DPPH and ABTS^+^ were directly related to the increment of chlorophylls, betacyanins, betaxanthins, betalains, and carotenoids or vice versa. It meant all studied phytopigments had good radical quenching ability. Similarly, ascorbic acid demonstrated a positive and significant interrelationship with TF, TP, and radical quenching ability in ABTS^+^ and DPPH, while it exhibited negative and insignificant correlations across all phytopigments. The earlier work of amaranth also reported a related trend [22, 26]. A positive and significant association was exhibited among β-carotene, ascorbic acid, TF, TP, and radical quenching ability in ABTS^+^ and DPPH. The significant positive interrelationship of β-carotene, ascorbic acid, TP, and TF and radical quenching ability in ABTS^+^ and DPPH signify that β-carotene, ascorbic acid, TP, and TF had a powerful activity of antioxidants. The capacity of antioxidants validation of amaranth through differential methods confirmed the significance in the associations between radical quenching ability in ABTS^+^ and DPPH. Studied phytopigments and phytochemicals including β-carotene, ascorbic acid, TP, and TF demonstrated powerful activity of antioxidants, as these showed the significant associations with radical quenching ability in ABTS^+^ and DPPH. All studied phytopigments, β-carotene, ascorbic acid, TP, and TF played a vital role in the antioxidant potentiality of the amaranth lines as these compounds had a powerful activity of antioxidants.

## Conclusions

The selected amaranth contained adequate nutraceuticals, phytopigments, phytochemicals including radical quenching ability. Across nine identified flavonoids, naringenin, apigenin, catechin, myricetin were newly reported in drought-tolerant vegetable amaranth. Based on this study, pharmacologists could investigate the suitability of these crops in medicinal drug discovery. Across the accessions, AT7 and AT15 had abundant phytochemicals and radical quenching ability including considerable nutraceuticals and phytopigments in comparison to the accessions AT3 and AT11. The accessions AT7 and AT15 might be directly utilized as nutraceuticals, phytopigments, phytochemicals enrich high yielding commercial cultivars. AT7 and AT15 could also be utilized to extract juice as a source of potential nutritional value, antioxidant phytopigments, β-carotene, antioxidants, flavonoids, phenolics, and ascorbic acid in the diet to accomplishing nutritional and antioxidant sufficiency in the globe. These lines might significantly contribute to the promotion of health benefits and feeding the community across the globe deficit in nutraceuticals and antioxidants. Identified flavonoid compounds in amaranth genotypes open the new route for details pharmacological study.

## Data Availability

All data generated or analyzed during this study are included in this article and available from the corresponding author on reasonable request.

## Abbreviations

ANOVA: Analysis of variance
ABTS^+^: 2,2′-azino-bis-(3-ethylbenzothiazoline-6-sulphonic acid)
AAS: Atomic absorption spectrophotometry
AOAC: Association of Official Agricultural Chemists
AsA: Ascorbic acid
DPPH: 2,2-diphenyl-1-picrylhydrazyl
DHA: Dehydroascorbate
DMRT: Duncan’s Multiple Range Test
DTT: Dithiothreitol
DW: Dry weight
FW: Fresh weight
FRAP: Ferric reducing antioxidant power
GAE: Gallic acid equivalent
HCl: Hydrochloric acid
HPLC-UV: High performance liquid chromatography-ultra violet
LC-MS-ESI: Liquid chromatography-mass spectroscopy-electrospray ionization
ORAC: Oxygen radical absorbance capacity
RCBD: Completely randomized block design
RE: Rutin equivalent
ROS: Reactive oxygen species
RQA: Radical quenching activity
SD: Standard deviation
TEAC: Trolox equivalent antioxidant capacity
TF: Total flavonoid content
TP: Total polyphenol content
